# Acute heart failure due to fulminant eosinophilic myocarditis with mononeuritis: eosinophilic granulomatosis with polyangiitis requiring cardiac multimodality imaging and systemic evaluation: a case report

**DOI:** 10.1093/ehjcr/ytaf010

**Published:** 2025-01-17

**Authors:** Jun-ichi Noiri, Hiroki Matsuzoe, Ryo Nishio, Youhei Fujiki, Kenichiro Otani, Mayumi Inaba, Hiroshi Takaishi, Hidekazu Tanaka

**Affiliations:** Department of Cardiovascular Medicine, Yodogawa Christian Hospital, 1-7-50 Kunijima, Higashi Yodogawa, 533-0024 Osaka, Japan; Division of Cardiovascular Medicine, Department of Internal Medicine, Kobe University Graduate School of Medicine, 7-5-1 Kusunoki, Chuo, 650-0017 Kobe, Japan; Department of Cardiovascular Medicine, Yodogawa Christian Hospital, 1-7-50 Kunijima, Higashi Yodogawa, 533-0024 Osaka, Japan; Division of Cardiology, Kindai University Faculty of Medicine, 377-2 Onohigashi, Osakasayama, 589-8511 Osaka, Japan; Department of Cardiovascular Medicine, Yodogawa Christian Hospital, 1-7-50 Kunijima, Higashi Yodogawa, 533-0024 Osaka, Japan; Department of Rheumatology, Yodogawa Christian Hospital, 1-7-50 Kunijima, Higashi Yodogawa, 533-0024 Osaka, Japan; Department of Respiratory Medicine, Yodogawa Christian Hospital, 1-7-50 Kunijima, Higashi Yodogawa, 533-0024 Osaka, Japan; Department of Pathology, Yodogawa Christian Hospital, 1-7-50 Kunijima, Higashi Yodogawa, 533-0024 Osaka, Japan; Department of Cardiovascular Medicine, Yodogawa Christian Hospital, 1-7-50 Kunijima, Higashi Yodogawa, 533-0024 Osaka, Japan; Division of Cardiovascular Medicine, Department of Internal Medicine, Kobe University Graduate School of Medicine, 7-5-1 Kusunoki, Chuo, 650-0017 Kobe, Japan

**Keywords:** Case report, Churg–Strauss syndrome, Eosinophilic myocarditis, Eosinophilic granulomatosis with polyangiitis, Endomyocardial biopsy, Heart failure, Multidisciplinary approach

## Abstract

**Background:**

Eosinophilic granulomatosis with polyangiitis (EGPA) is a systemic vasculitis that affects small-to-medium vessels of various organs and can lead to eosinophilic myocarditis, a rare but life-threatening condition. The diagnosis of EGPA is challenging due to overlapping features with other forms of vasculitis. Additionally, various clinical presentations of EGPA make its management complicated.

**Case summary:**

A 55-year-old man with a history of asthma presented with worsening chest pain. Peripheral hyper-eosinophilia, elevated troponin level, refractory pulseless ventricular tachycardia, and severe cardiac dysfunction suggested fulminant eosinophilic myocarditis. A multidisciplinary team comprising rheumatology, respirology, haematology, pathology, and cardiology specialists discussed the underlying cause of eosinophilia and diagnosed EGPA with the pathological findings of endomyocardial biopsy (EMB). Immunosuppressive therapy and optimal medical therapy for acute heart failure resulted in remission of myocarditis, as confirmed by follow-up echocardiography, cardiac magnetic resonance imaging, and EMB. Despite a good clinical course, mononeuritis rapidly worsened just before his discharge, requiring additional therapy. During the 2-year outpatient follow-up, the cardiac function remains well, and mononeuritis also improved.

**Discussion:**

The multidisciplinary approach facilitated prompt and accurate diagnosis and treatment, despite the disease’s diverse presentation, ultimately saving the patient’s life. This case highlights the importance of systemic evaluations in patients with EGPA, potentially affecting multiple organs, for monitoring disease status and guiding its treatment. This case report also emphasizes that close follow-up and careful monitoring with cardiac multimodality imaging are important to ensure optimal management of heart failure caused by EGPA myocarditis.

**Learning points**

- Eosinophilic granulomatosis with polyangiitis (EGPA) potentially develops fulminant eosinophilic myocarditis, and its diagnosis is challenging due to its rarity, varied clinical presentation, and overlapping features with other forms of vasculitis; hence, necessitating a multidisciplinary approach.
- Eosinophilic granulomatosis with polyangiitis can evolve into three different phases with various symptoms and requires thorough systemic evaluation to identify all sites of disease involvement to significantly improve prognosis and reduce morbidity and mortality in these patients.
- Close follow-up and careful monitoring with cardiac multimodality imaging are important to ensure optimal management of heart failure caused by EGPA myocarditis.

## Introduction

Eosinophilic granulomatosis with polyangiitis (EGPA), formerly known as Churg–Strauss syndrome, is a systemic, small-to-medium vessel necrotizing vasculitis characterized by asthma, eosinophilia, and eosinophil infiltration of various organs.^1^ Cardiac involvement is rare and associated with poor prognosis, accounting for ∼50% of EGPA-attributable deaths,^2^ and includes symptoms such as heart failure, intracardiac thrombi, myocardial ischaemia, arrhythmias, and myocarditis.^3,4^ Rapid diagnosis and systemic glucocorticoid immunosuppressant agents improve patient prognosis, but EGPA diagnosis is challenging. Furthermore, evaluation of therapeutic effectiveness for EGPA-associated myocarditis is difficult due to the lack of reliable biomarkers and imaging examinations to measure disease activity and organ involvement. Herein, we report the diagnostic and management challenges associated with fulminant eosinophilic myocarditis and mononeuritis due to EGPA, emphasizing the importance of comprehensive cardiac imaging and systemic evaluation.

## Summary figure

**Table:** 

Time point	Event
12 years prior (43 years old)	First diagnosis of asthma.
2 months prior	Worsening dyspnoea and rhinorrhoea.
1 week prior	Appearance of chest discomfort.
2 days prior	Diagnosis of exacerbation of chronic sinusitis.
Day of admission	Worsening chest pain.
	Twelve-lead electrocardiogram: a new-onset incomplete left bundle branch block with ST-segment depression in leads V4–6. Laboratory exams: high-sensitive troponin I (15 182.4 pg/mL), brain natriuretic peptide (315.8 pg/mL), C-reactive protein (134 mg/L), eosinophils (12.18 × 10^9^/L). Transthoracic echocardiogram (TTE): left ventricular ejection fraction (LVEF) 32.9%, global longitudinal strain (GLS) −3.3%, pericardial effusion with right ventricular collapse, and moderate mitral regurgitation. Coronary angiography: normal coronary.
	Ventilator management was needed due to acute heart failure. Administered intravenous methylprednisolone and normalization of eosinophilic count.
Within the first week of hospitalization	Cardiac magnetic resonance (CMR): diffuse late gadolinium enhancement (LGE). Endomyocardial biopsy (EMB): diffuse infiltration of eosinophils, necrosis, and fibrosis of myocardium. Depending on diagnosis of eosinophilic granulomatosis with polyangiitis, immunosuppressive therapy was initiated. Up-titration of guideline-directed medical therapy for heart failure with reduced ejection fraction.
On the second week of hospitalization	Improvement of eosinophilia and cardiac function was observed. TTE: LVEF 53.6%, GLS −16.4%. CMR: decreased LGE area.
37th day of hospitalization	Weakness of the left tibialis anterior and extensor hallucis longus muscles rapidly became worse. Nerve conduction study: conduction block in the peroneal nerve.
	EMB: remission of eosinophilic infiltration.
Discharge (Day 49)	Administration of mepolizumab for mononeuritis.
Present (two years after the discharge)	No episode of heart failure or relapse of EGPA. TTE: LVEF 53.8%, GLS −13.7%.

## Case presentation

**Figure 1:**
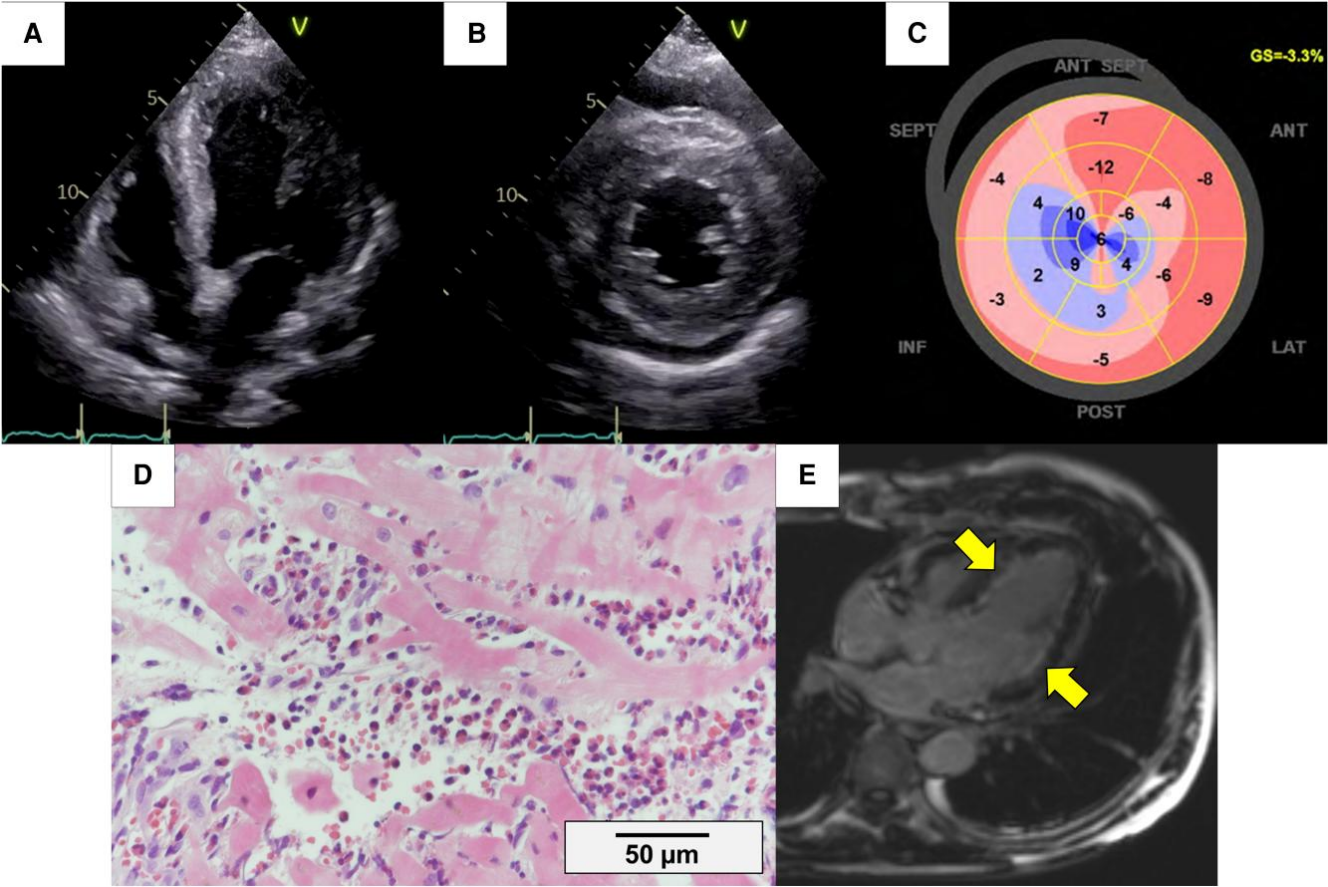
Main findings in the acute phase. (*A* and *B*) TTE showing myocardial thickening and pericardial effusion. (*C*) GLS of −3.3%. (*D*) EMB (haematoxylin and eosin staining, bar denotes 50 μm) showing scattered necrotic and fibrotic myocardium with diffuse eosinophilic infiltration. (*E*) CMR images showing widespread subendocardial LGE (arrows). CMR, cardiac magnetic resonance imaging; EMB, endomyocardial biopsy; TTE, transthoracic echocardiogram; GLS, global longitudinal strain; LGE, late gadolinium enhancement.

A 55-year-old man presented to our hospital due to worsening chest pain, preceded by dyspnoea and rhinorrhoea for the past 2 months and chest discomfort for 5 days. He was diagnosed with bronchial asthma, chronic sinusitis, and allergic rhinitis 12 years before presentation, hospitalized four times for status asthmaticus, and had an uneventful course over the last 6 years. Physical examination revealed an afebrile status, gallop rhythm on heart sound, jugular vein distention, saddle nose, palpable purpura on the lower extremities, and no neurological signs. Laboratory tests revealed evidence of an inflammatory state with marked hyper-eosinophilia [C-reactive protein (normal range: <5 mg/L): 134 mg/L, white blood cell count (normal range: 4.0–10.0 × 10⁹/L): 20.3 × 10⁹/L, eosinophils (normal range: 0.0–0.5 × 10⁹/L): 12.18 × 10⁹/L, 60% of leucocytes], myocardial injury [high-sensitivity troponin I (normal range: <20 ng/L): 15 182.4 ng/L], negative anti-neutrophil cytoplasmic antibodies (ANCAs), and an elevated brain natriuretic peptide level [BNP (normal range: <18.4 pg/mL): 315.8 pg/mL]. An electrocardiogram showed a new-onset incomplete left bundle branch block that was absent 6 years prior (see Supplementary material online, *Figure S1*). Chest radiography demonstrated bilateral congestion. Transthoracic echocardiogram (TTE) revealed severe left ventricular systolic dysfunction [left ventricular ejection fraction (LVEF): 32.9%, global longitudinal strain (GLS): −3.3%] with myocardial thickening and pericardial effusion (*Figure 1A–C*, Supplementary material online, *Video S1*). Coronary angiography showed no significant stenosis, excluding acute coronary artery disease. A multidisciplinary team comprising rheumatology, respirology, haematology, pathology, and cardiology specialists convened to discuss the cause of the eosinophilia (*Table 1*). The patient had no history of exposure to new medications that might have triggered hypersensitivity. Lymphoid and myeloid neoplasms were ruled out based on findings from bone marrow aspiration and molecular cytogenetic analyses. Results from immunological and allergy testing, including antinuclear antibodies and immunoglobulin levels, were inconclusive. Comprehensive serological testing was conducted to exclude viral and parasitic infections, with all results returning negative. This multidisciplinary evaluation raised the possibility of EGPA as a potential diagnosis. To confirm the diagnosis, biopsies were obtained from the right ventricular septum and skin.

**Table 1:** Differential diagnosis of eosinophil-associated diseases and disorders

Immunologic disorders
Eosinophilic granulomatosis with polyangiitis
Giant cell arteritis
Polyarteritis nodosa
Sarcoidosis
IgG4 disease
Allergic bronchopulmonary aspergillosis
Idiopathic hyper-eosinophilic syndrome
Immunodeficiencies (e.g. hyper-IgE syndrome and Omenn syndrome)
Hypersensitivity disorders
Drug reaction with eosinophilia and systemic symptoms
Eosinophilia-myalgia syndrome
Interstitial nephritis
Infectious diseases
Helminths (e.g. strongyloidiasis and trichinellosis)
Ectoparasites (e.g. scabies and myiasis)
Protozoans (e.g. isosporiasis and sarcocystis myositis)
Fungi (e.g. coccidiomycosis and histoplasmosis)
Viral (e.g. HIV)
Neoplastic disorders
Primary hyper-eosinophilic syndromes (e.g. *FIP1L1-PDGFRA* rearrangement)
Acute or chronic eosinophilic leukaemia
Other myeloid neoplasms (e.g. chronic myeloid leukaemia and systemic mastocytosis)
Lymphoid malignancies (e.g. T- and B-cell lymphomas)
Solid tumours (e.g. adenocarcinoma and squamous carcinoma)
Pregnancy-related
Idiopathic/undefined

*FIP1L1-PDGFRA*, FIP1-like-1-platelet-derived growth factor receptor alpha; HIV, human immunodeficiency virus; IgE, immunoglobulin E; IgG4, immunoglobulin G4.

Considering EGPA, methylprednisolone (1000 mg/day) was initiated on admission, which led to normalization of the eosinophil count the following day. On the sixth hospital day, the pathological findings of endomyocardial biopsy (EMB) from the right ventricular septum revealed diffuse eosinophil infiltration, myocardial necrosis, and fibrosis (*Figure 1D*), confirming eosinophilic myocarditis caused by EGPA. Cyclophosphamide (1000 mg/day) and intravenous immunoglobulin (400 mg/kg for 5 days) were immediately administered thereafter, considering the severity of fulminant myocarditis. The eosinophil counts stabilized, pulmonary congestion improved, and pulseless ventricular tachycardia resolved. Cardiac magnetic resonance (CMR) imaging showed significant left ventricular subendocardial late gadolinium enhancement (LGE) (*Figure 1E*). Skin biopsy revealed lymphocytic infiltration around the capillaries and eosinophilic infiltration in the subcutaneous fatty tissue, supporting the EGPA diagnosis.

**Figure 2:**
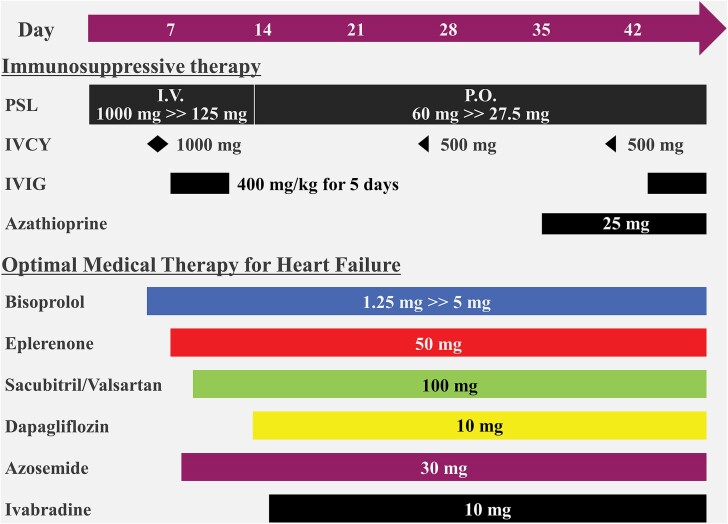
Immunosuppressive therapy and optimal medical therapy for heart failure. The patient was treated with prednisolone (PSL), intravenous cyclophosphamide (IVCY), intravenous immunoglobulin (IVIG), and azathioprine as immunosuppressive therapy. Additionally, optimal medical therapy for heart failure with reduced ejection fraction was administered and up-titrated with bisoprolol, eplerenone, sacubitril/valsartan, dapagliflozin, azosemide, and ivabradine. I.V., intravenous; P.O., per os.

**Figure 3:**
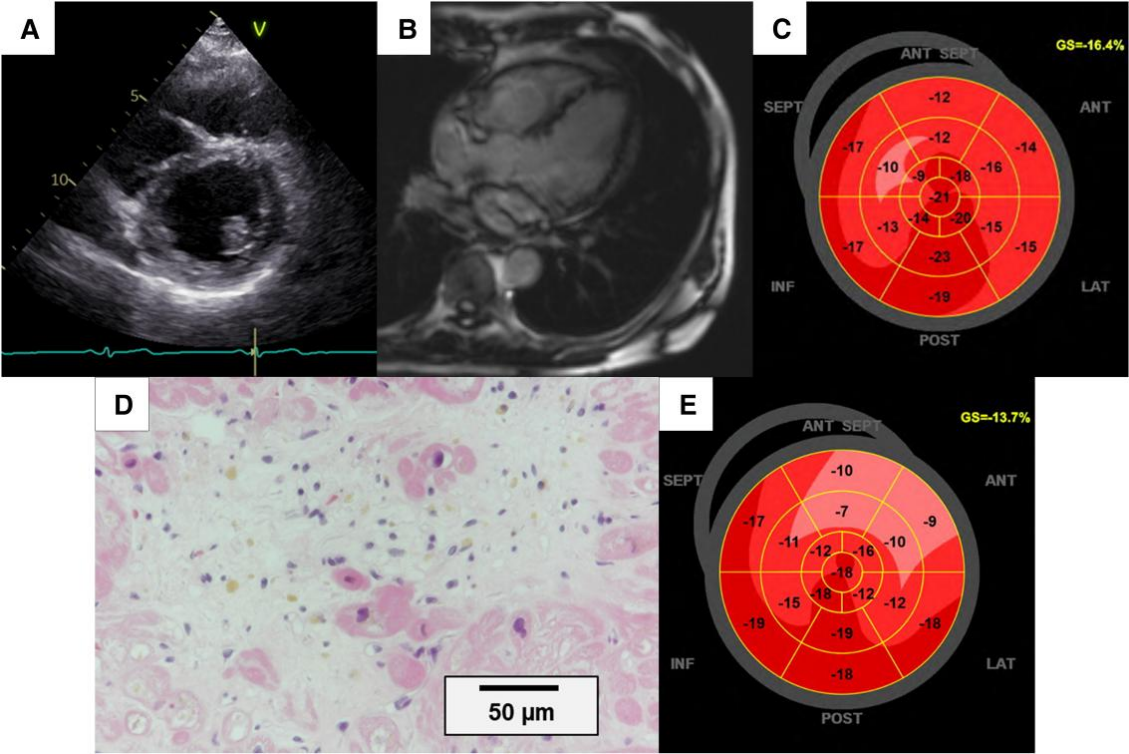
Imaging evaluations in the late phase. (*A*) TTE before discharge showing improvement in left ventricular thickness. (*B*) CMR disappearance of late gadolinium enhancement. (*C*) GLS is also improved: −16.4%. (*D*) EMB (haematoxylin and eosin stain, bar denotes 50 μm) showing remission of eosinophilic infiltration. (*E*) Impaired GLS remained at 2-year follow-up: −13.7%. CMR, cardiac magnetic resonance imaging; EMB, endomyocardial biopsy; TTE, transthoracic echocardiogram; GLS, global longitudinal strain.

Additional EGPA-directed treatment with oral corticosteroids, azathioprine, and cyclophosphamide led to a good clinical course (*Figure 2*). On the 14th day of admission, TTE showed improved cardiac function (LVEF: 53.6%, GLS: −16.4%) (*Figure 3A* and *C*, Supplementary material online, *Video S2*). Cardiac magnetic resonance showed decreased LGE area (*Figure 3B*) and eosinophilic stabilization.

Besides investigating the heart, systemic evaluation (physical examination for purpura and neuropathy, chest radiography for lung involvement, faecal occult blood test for gastroenteritis, and urinalysis for renal failure) was repeatedly performed. Despite a favourable clinical course, gradual weakness of the left tibialis anterior and extensor hallucis longus muscles emerged around Day 20, deteriorating by Day 32 although additional intravenous cyclophosphamide was administered. A nerve conduction study revealed a conduction block in the peroneal nerve, suggesting mononeuritis. A multidisciplinary team considered EGPA relapse and decided to re-examine EMB from the right ventricular septum and administer additional immunosuppressive therapy, including azathioprine (25 mg/day) and immunoglobulin. Although the steppage gait did not improve, the pathological absence of ongoing myocarditis (*Figure 3D*) prompted the patient’s discharge on Day 49 with administration of mepolizumab, an anti-interleukin-5 monoclonal antibody, for mononeuritis.

The patient is currently receiving prednisolone, azathioprine, mepolizumab, and guideline-directed medical therapy for EGPA and heart failure. He had no episode of heart failure rehospitalization or EGPA relapse for 2 years. His cardiac function was preserved as shown on TTE; therefore, a cardioverter-defibrillator was not implanted. Furthermore, the mononeuritis improved gradually.

## Discussion

Eosinophilic granulomatosis with polyangiitis can cause rare and serious eosinophilic myocarditis, which needs to be differentiated from other conditions, including hypersensitivity, hyper-eosinophilic syndrome, infection, pregnancy-related issues, malignancy, toxic causes, and idiopathic origins.^5^ Recently, new classification criteria have been proposed (*Table 2*).^6^ Asthma is a major diagnostic criterion for EGPA but is often overlooked in patients with EGPA because cardiologists may be unfamiliar with the condition and its diagnostic criteria.^5^ Therefore, a multidisciplinary approach is recommended to facilitate prompt and accurate diagnosis. Cardiac damage in EGPA primarily results from eosinophilic infiltration and vasculitis. Activated eosinophils infiltrate the myocardium and release toxic granule proteins that damage cardiomyocytes and cause vasculitis, resulting in coronary artery spasm and myocardial infarction. Endomyocardial biopsy and CMR are useful in diagnosing patients with suspected cardiac lesions due to EGPA (*Table 3*).^7^

**Table 2:** 2022 ACR/EULAR classification criteria for EGPA

Clinical criteria	
Obstructive airway disease	+3
Nasal polyps	+3
Mononeuritis multiplex	+1
Laboratory and biopsy criteria	
Blood eosinophil count ≥ 1×10^9^/L	+5
Extravascular eosinophilic-predominant inflammation on biopsy	+2
Positive test for c-ANCA or anti-PR3 antibodies	−3
Haematuria	−1

Calculate the sum of the scores for all seven items. A score of ≥6 is required for the classification of EGPA.

ACR/EULAR, American College of Rheumatology/European Alliance of Associations for Rheumatology; c-ANCA, cytoplasmic anti-neutrophil cytoplasmic antibody; EGPA, eosinophilic granulomatosis with polyangiitis; PR3, proteinase 3.

**Table 3:** Examinations for the screening, diagnosis, and follow-up of EGPA

	Screening	Diagnosis	Follow-up
Physical examination	○	○	○
Laboratory test	○		
ECG	○		
TTE	○	○	○
CMR			○
EMB		○	○

CMR, cardiac magnetic resonance imaging; ECG, electrocardiography; EGPA, eosinophilic granulomatosis with polyangiitis; EMB, endomyocardial biopsy; TTE, transthoracic echocardiography.

**Figure 4:**
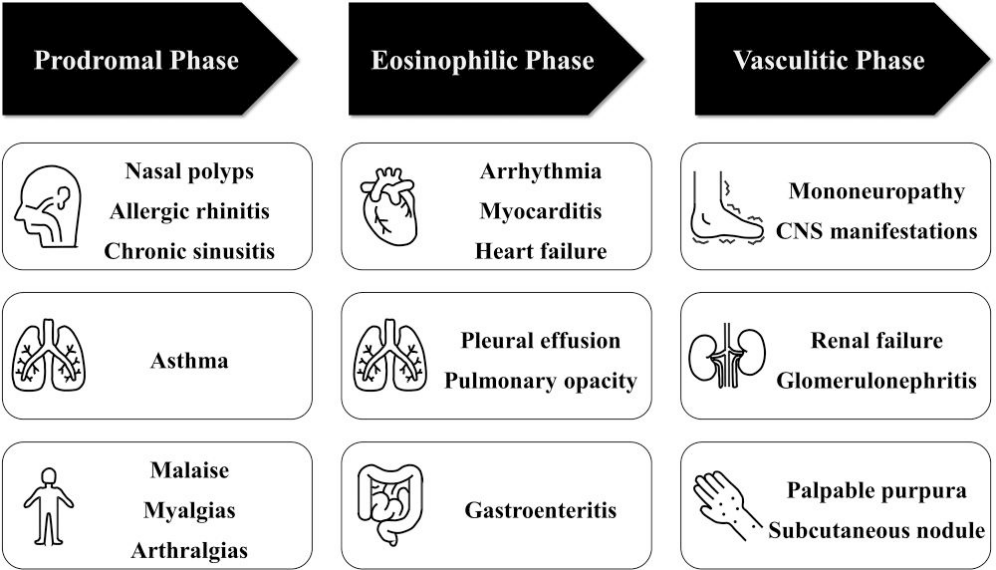
Three consecutive phases of EGPA. EGPA evolves through prodromic, eosinophilic, and vasculitis phases. CNS, central nervous system; EGPA, eosinophilic granulomatosis with polyangiitis.

Eosinophilic granulomatosis with polyangiitis usually progresses in three phases (*Figure 4*) that often overlap. Patients with cardiac involvement in the eosinophilic phase should be monitored for cardiac symptom relapse and vasculitis symptoms using a multidisciplinary approach. Our patient showed improvement in myocarditis on TTE, CMR, and EMB while developing neuropathy symptoms. This discrepancy in cardiac and neurological findings highlights the importance of systemic evaluation. Peripheral neuropathy, usually mononeuritis, often develops in patients with EGPA (51.4%).^8^ Neuropathy is a severe complication of EGPA that leads to muscle atrophy and neuropathic pain. Mepolizumab is an effective and safe add-on therapy in patients with relapsing or refractory EGPA^9^ with EGPA-induced neuropathy.^10^ Early recognition of mononeuritis and additional treatment with mepolizumab possibly improved our patient’s outcome.

Eosinophilic granulomatosis with polyangiitis typically relapses even after remission. Notably, cardiac involvement is a predictor of frequent relapse; accordingly, patients with myocarditis due to EGPA require careful monitoring.^11^ However, no reliable biomarkers have been established to detect EGPA relapse. The latest evidence-based guidelines mention that EGPA should be monitored clinically by detecting signs and symptoms and performing imaging studies.^7^ Periodic TTE and CMR are recommended in all patients with EGPA under treatment for the early detection of cardiac involvement relapse.^12^ Cardiac magnetic resonance results reportedly correlate with EMB findings.^13^ However, the effectiveness of CMR in detecting EGPA myocarditis relapse remains unclear.^14^ Despite being invasive, clinical guidelines recommend EMB for suspected EGPA myocarditis in critical conditions to assess the need for immunosuppressive therapy.^7^ Hence, we determined that a detailed evaluation of myocarditis relapse was crucial for considering more intensive immunosuppressive therapy.

Eosinophilic granulomatosis with polyangiitis clinical guidelines recommend adjusting remission-induction treatment based on prognostic factors.^7^ For organ-threatening manifestations, such as cardiomyopathy, renal insufficiency, and CNS involvement, EMB is advised to determine the need for immunosuppressive therapy. Patients with poor prognostic factors should receive pulsed intravenous glucocorticoids (500–1000 mg of methylprednisolone daily for 3 days), followed by high-dose oral glucocorticoids (0.75–1 mg/kg per day). In severe cases, cyclophosphamide is added until remission (every 2 weeks for 1 month, then every 4 weeks at 0.6 g/m² per dose). Rituximab (1 g pulses every 2 weeks) is also effective. For refractory EGPA, intravenous immunoglobulin (400 mg/kg daily for 5 days) may be considered. For maintenance, rituximab, mepolizumab, or azathioprine with glucocorticoids is recommended. Glucocorticoids should be tapered to the minimum effective dose. Rheumatologists should determine azathioprine dosage considering the limited information on its appropriate dosage. We used pulsed intravenous glucocorticoids and cyclophosphamide for remission induction and added intravenous immunoglobulin for refractory myocarditis. For maintenance, we tapered oral glucocorticoids and added azathioprine (25 mg per day) as per the rheumatologist’s advice (*Figure 2*).

For acute heart failure, the European Society of Cardiology Guidelines recommend initiation and rapid up-titration of evidence-based treatment before discharge and during frequent and careful follow-up visits to reduce the risk of heart failure, rehospitalization, or death.^15^ Our patient showed good clinical course per the guideline-directed medical therapy and close follow-up. Notably, GLS worsened regardless of the preserved LVEF during the 2-year follow-up (*Figure 3E*, Supplementary material online, *Video S3*). Global longitudinal strain is also an independent predictor of adverse outcomes in patients with heart failure with preserved ejection fraction.^16^ Eosinophilic granulomatosis with polyangiitis-associated myocarditis can cause long-term myocardial damage, including restrictive or dilated cardiomyopathy, both independent risk factors for mortality^17^; thus, GLS may be useful in predicting the progression to cardiac dysfunction and adverse events during the follow-up of EGPA-related myocarditis. In our case, although the eosinophil counts normalized and myocardial eosinophilic infiltration improved, GLS decreased and mononeuritis persisted. These findings may indicate ongoing EGPA activity. Previous reports on EGPA-associated myocarditis have primarily focused on cardiac involvement assessed via CMR.^18^ To the best of our knowledge, few studies have investigated the role of GLS evaluation following EGPA-associated myocarditis or explored the relationship between cardiac and extracardiac involvement. Consequently, further research is needed to explore these associations. Nevertheless, this case underscores the importance of using GLS to assess subclinical myocardial damage and highlights the need for continuous systemic evaluation in EGPA-related myocarditis, even in the absence of overt clinical signs of cardiac involvement.

In conclusion, EGPA-associated myocarditis is fatal and requires early diagnosis and prompt treatment. Systemic evaluation and EMB are essential for obtaining an accurate diagnosis. Since EGPA progresses through three stages, systemic evaluation is crucial to detect organ involvement during treatment. Cardiac multimodal imaging throughout the acute and chronic phases is important for monitoring disease status and guiding treatment.

## Supplementary Material

ytaf010_Supplementary_Data

## Data Availability

Data sharing is not applicable to this article, as no datasets were generated or analysed during the current study. The data underlying this article are available in the article and online Supplementary material.

## Lead author biography

Dr Jun-ichi Noiri is a cardiologist at Kobe University Graduate School of Medicine, Japan. He completed his medical training at Hiroshima University and residencies at Yodogawa Christian Hospital and National Cerebral and Cardiovascular Center. His expertise lies in valvular heart disease, cardiomyopathy, pulmonary circulation, and heart failure. He is currently an active researcher and clinician, with a primary focus on echocardiography.

## Supplementary material

**Consent:** The authors confirm that written consent for submission and publication of this case including images and associated text has been obtained from patient in line with COPE guidance.

**Funding:** The authors received no external funding or financial support.
