# Supplementary figures and images for: Acute heart failure due to fulminant eosinophilic myocarditis with mononeuritis: eosinophilic granulomatosis with polyangiitis requiring cardiac multimodality imaging and systemic evaluation: a case report
Source: Eur Heart J Case Rep. 2025 Jan 17;9(2):ytaf010. doi: 10.1093/ehjcr/ytaf010 (PMC11804243; doi:10.1093/ehjcr/ytaf010)

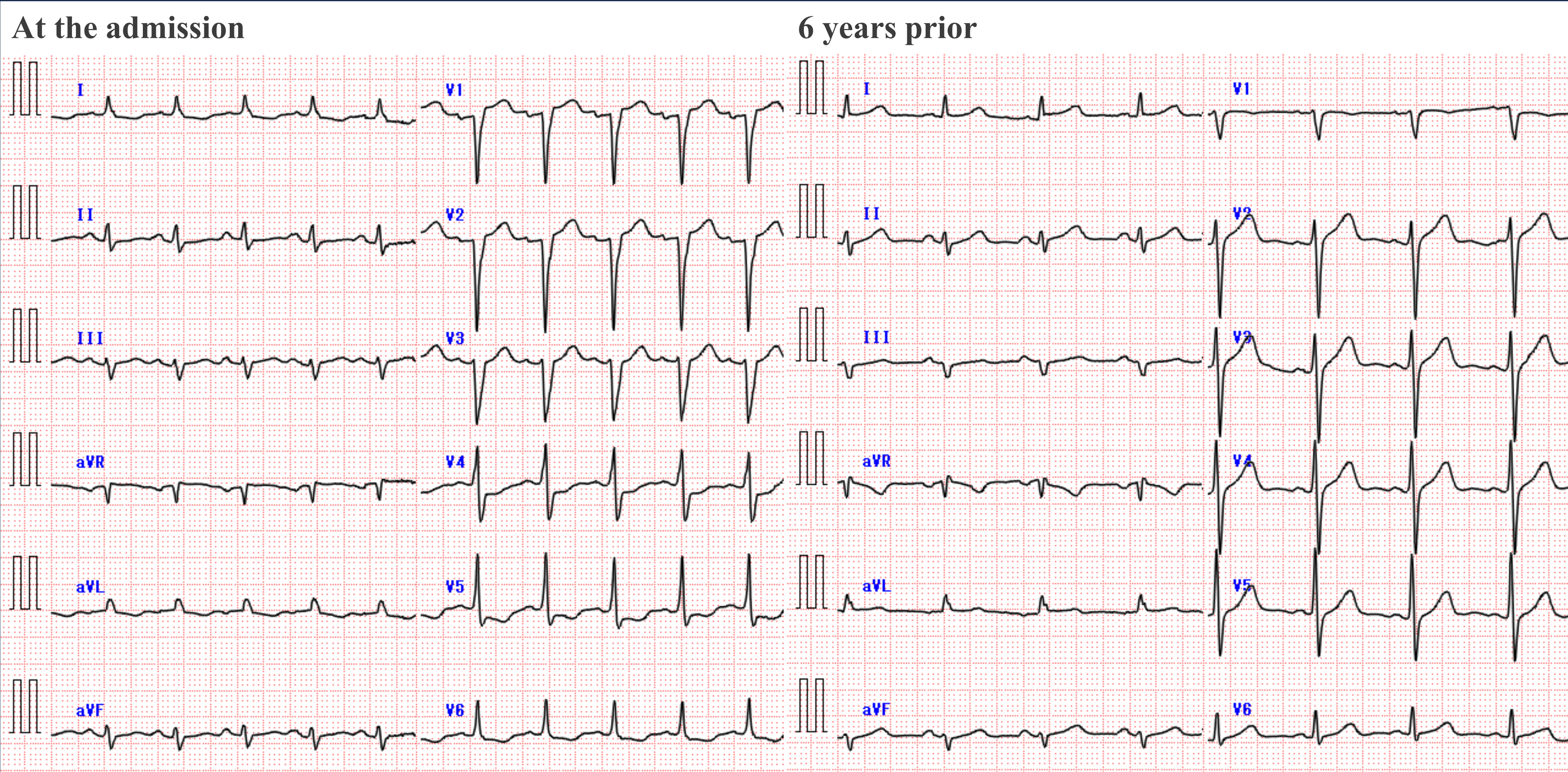

Supplement: ytaf010_Supplementary_Data [file ytaf010_supplementary_data.zip › FigureS1 (1).tif]
